# Investigation of Death Due to Fever in Patrasayer Block in the District of Bankura, West Bengal

**DOI:** 10.4103/0970-0218.58410

**Published:** 2009-10

**Authors:** Nirmal Kumar Mandal, Dipta Kanti Mukhopadhyay, Asit Baran Saren, Tanmay Kanti Panja, Nirmalya Sinha, Akhil Bandhu Biswas

**Affiliations:** Department of Community Medicine, B.S. Medical College, Bankura. E-mail: E-mail: nirmalbrp@yahoo.co.in

Sir,

Communicable diseases contribute to a significant disease burden and are a major cause of morbidity, mortality, and long-term, severe mental and physical disabilities.(1) Disease surveillance is needed to recognize cases or a cluster of cases, to identify a high-risk group or geographic areas, with a view to initiate an effective response in a timely manner, to prevent transmission of disease or to reduce morbidity and mortality.(2)

In the case of the death due to fever of unknown origin of two persons in the Patrasayer block of Bankura District, West Bengal, an investigation was conducted on the request of the District Health Officials, to investigate the deaths to find a suggestive cause of death and to suggest the control measures to mitigate the situation and to prevent future occurrence of such incidents.

The team visited Birsingha and Champabani villages in the Patrasayer Block on 2 April, 2008, and the sequence of events that occurred in the lives of the deceased persons up to their death were collected from close relatives, by verbal autopsy. Bed head tickets, other records, and reports related to these deceased persons were reviewed. Information was collected from Medical Officers (MOs), Public Health Nurses (PHNs), Auxiliary Nurse Midwives (ANMs), and Medical Technologists.

The index case was a 22-year-old male, a daily laborer of Birsingha village of Patrasayer Block. He and his 20-year-old wife visited his in law's house at Chapaboni village of same Block. He had a low-grade fever with malaise for three days. His wife was pregnant, in the third trimester, with jaundice. Unexpectedly, both of them developed high intermittent fever with rigor and intense headache, and after three days both had altered sensorium, followed by unconsciousness, without any history of convulsion. They were initially managed conservatively at Sonamukhi Rural Hospital and recorded to have nuchal rigidity. The attending doctor reached a clinical diagnosis of meningo-encephalitis, not confirmed by laboratory investigation. Blood slides, 276, from the same two villages were taken from the inhabitants surrounding the household of the two deceased persons and examined. They were found to be negative for malaria parasite. Ten percent of the slides were randomly taken by the investigating team and cross-checked at B.S. Medical College, Bankura, and found to be negative for malaria parasite. No case of Plasmodium falciparum was reported in the last three years at these localities.

On query, it was evident that death due to similar clinical presentation of intermittent high rise in temperature for three to five days, with headache and malaise, followed by unconsciousness, had taken place earlier in both villages. In the Chapaboni village, with a population of 438, deaths of two male persons aged between 20 and 25 years were reported in the last two years. In Birsingha village with a population of 5490, a couple within the age group of 30 - 40 years had expired within the last eight months, as a consequence of similar disease manifestations. Causes of these deaths were not confirmed by laboratory test.

Patrasayer Block is a bordering area with Burdwan District, endemic for Japanese encephalitis, having similar environmental conditions, such as, vast irrigated paddy fields encircling the human habitats, plenty of water-land attracting several types of migrating birds, rearing pigs, and cattle as common domestic animals living closely with human beings.

Two couples died with same type of fever of short duration. So, man to man transmission could not be ruled out. However, no laboratory evidence was available to label it as bacterial meningitis. Ecological background, male predilection, sporadic distribution, and clinical presentation like fever with impaired sensorium and unconsciousness went in favor of vector borne viral encephalitis (JE).

Following recommendations were made:

- Any case of fever and altered sensorium should be suspected to be JE. It should be immediately notified to the higher authorities for further action.
- Spot mapping of fever cases with loss of sensorium, in the block along with adjacent blocks (retrospectively for at least for last two years, as well as, prospectively) is recommended, to specify place distribution of the disease.
- Entomological survey is required to detect the species of the vector.
- Proper investigation of such cases reported to any health facilities should be ensured, especially analysis of Blood (Pf and PV Ag, Blood slides for malaria parasite) and Cerebrospinal fluid, for confirmation of meningitis and serum for JE.
